# Microwave-Reactor-Based Preparation of Red Iron Oxide Pigment from Waste Iron Sulfate

**DOI:** 10.3390/ma16083242

**Published:** 2023-04-20

**Authors:** Kamila Splinter, Dariusz Moszyński, Zofia Lendzion-Bieluń

**Affiliations:** Department of Inorganic Chemical Technology and Environment Engineering, Faculty of Chemical Technology and Engineering, West Pomeranian University of Technology in Szczecin, Piastów Ave. 42, 71-065 Szczecin, Poland

**Keywords:** iron red, solid waste, hematite, waste iron sulfate, iron pigment

## Abstract

This article presents a two-step method of iron red synthesis based on waste long-term deposited iron(II) sulfate. The first step is the purification of waste iron sulfate, and then the pigment is synthesized by precipitation using a microwave reactor. The newly developed method of purification allows for quick and thorough purification of iron salt. The use of a microwave reactor in the synthesis of iron red makes it possible to reduce the temperature of the goethite–hematite phase transition from 500 °C to 170 °C and skip the calcination process. A temperature reduction in the synthesis decreases the formation of agglomerates of synthesized materials compared to commercial ones. The results of the research showed a change in the physicochemical properties of the obtained pigments depending on the conditions of synthesis. Waste iron(II) sulfate is a promising raw material for the synthesis of iron red pigments. Laboratory pigments are found to be differ from commercial pigments. The difference in properties speaks in favor of synthesized materials.

## 1. Introduction

As far as iron pigment manufacturing is concerned, precipitation and Penniman Zoph processes are the most commonly used procedures [1]. Using either process, there can be pigments produced with a wide range of colors that are associated with iron oxides and oxide–hydroxides. In this way, with minor process modifications, yellow, orange, red, and black pigments can be synthesized.

The main raw material that is used in both a precipitation and the Penniman–Zoph process is iron(II) sulfate. It is also relevant to note that the Penniman–Zoph procedure also takes into account the use of waste sulfate, e.g., from the production of TiO_2_ or from steel etching [2]. It is critical for this raw material, however, to be relatively fresh, nonoxidized, and free of any colored metal add-ons such as chromium or manganese compounds. The presence of additional colored oxides has a negative effect on the color of the pigment.

A natural development of the existing technologies [1,3] is to use chemical waste and check whether the obtained products are different from those made from pure raw materials. Chemical waste that can be used for the synthesis of iron pigments in accordance with the concept of “waste to materials” is increasingly being sought. The publication [4] uses iron sand from Dlodo beach in Tulungangung on the island of Java to obtain yellow and red pigments. The authors of the publication [5] derived coke from bituminous shale for the synthesis of hybrid red iron pigments. Waste from the production of stainless steel was repurposed by the authors of the article [6]. They used oily mill scale, which is a byproduct of hot rolling steel. The authors of [7,8] investigated the hydrochemical conversion of goethite (FeOOH) to magnetite (Fe_3_O_4_) in high-iron Bauxite residue. The iron-rich residues can be used in the steel industry or as a pigment. In the Chinese patents [9,10], the method for obtaining iron pigments from waste iron sulfate was developed. However, the synthesized pigments were obtained by calcining the resulting suspensions at 600–750 °C. The authors of [11] used cans from condensed milk to obtain iron(II) sulfate and then red pigments based on hematite. In addition, an interesting developing field is transparent iron pigments. They can be used for coloring packaging or impregnations for wooden surfaces—the carrier medium acquires the color of a given pigment while remaining transparent [12,13,14,15,16,17].

In the Polish patent [18], the method for obtaining sulfur dioxide from iron(II) sulfate heptahydrate can be applied instead of the current method of burning elemental sulfur to obtain sulfur dioxide in the production of sulfuric acid. Another potential use for waste iron sulfate is in battery technology. There have been a number of methods developed [19,20,21] for the production of LiFePO_4_ which can be used as a cathode material in lithium-ion batteries. Iron(II) sulfate is also used in water purification [22,23,24] or chromium(VI) reduction in cement [25,26]. However, the salt must be stripped of most impurities.

As of now, several technologies have been developed that make use of waste iron sulfate. However, they rely on green salt, which is relatively fresh salt, not oxidized. The aim of the research was to investigate the possibility of the synthesis of red iron pigments based on waste iron(II) sulfate (waste FeSO_4_) from the production of titanium dioxide using the sulfate method at Grupa Azoty Zakłady Chemiczne “POLICE” S.A. (GA POLICE), which was deposited in the years 1976–2012 in a landfill. The waste FeSO_4_ landfill covers an area of approximately 43 ha, with over 4 million tons of waste accumulated. Waste FeSO_4_ is separated at the stage of crystallization in the form of the so-called green salt, which, in addition to iron ions, also contains ions of other metals such as aluminum, manganese, chromium, and nickel. Due to a number of pollutants in the collected waste FeSO_4_, salt cannot be used and is a large environmental problem. Waste FeSO_4_ is deposited in an open area, so it is exposed to the effects of weather conditions, mainly rainfall. Acid leachate from the landfill can contaminate groundwater and reduce the diversity of flora and fauna within a few kilometers of the landfill.

This study developed a new purification method for waste FeSO_4_, and then a method for synthesizing red iron pigments using purified FeSO_4_. The optimal synthesis conditions for red iron pigments were explored in preliminary research [27,28]. By managing the landfill through the use of waste FeSO_4_, iron pigments can be produced, thereby restoring the environmental diversity of the area and making a significant contribution to the Sustainable Development Goals (Goals 9 and 12) [29], improving the environmental image of the company and meeting the growing demand for iron pigments.

## 2. Materials and Methods

### 2.1. Materials

Waste FeSO_4_ from GA POLICE was used to obtain iron oxides subjected to the purification process. Reagent-purity substances were also used: 10 wt% sulfuric acid (Chempur, Piekary Śląskie, Poland), 25 wt% ammonia water (Chempur, Piekary Śląskie, Poland), and 30 wt% hydrogen peroxide (Chempur, Piekary Śląskie, Poland). To compare the properties of the materials obtained, commercial pigments from four different manufacturers were also tested (Boruta Zachem, Bydgoszcz, Poland; Chempro, Białystok, Poland; Precheza, Přerov, Czech Republic; Edan, Kraków, Poland).

### 2.2. Purification of Waste FeSO_4_

The preparation of iron pigments from waste FeSO_4_ requires prior purification of insoluble components and doped metal ions. The work uses a developed method of purifying waste FeSO_4_ by recrystallization [30].

**Figure 1:**
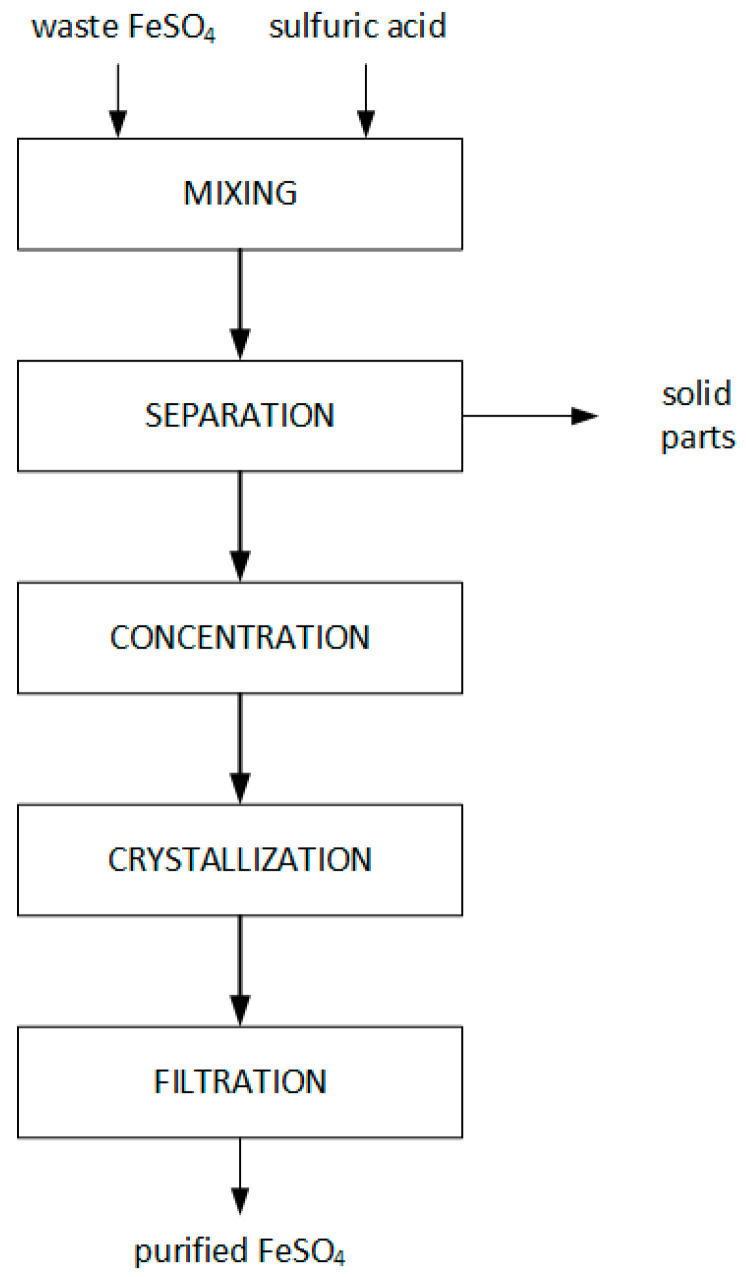
Scheme of the waste FeSO_4_ purification process.

For purification, a saturated waste FeSO_4_ solution in 10% sulfuric acid was prepared. A total of 130 g of waste FeSO_4_ was weighed and dissolved in 400 mL of 10% sulfuric acid in room temperature. The solution was centrifuged (1500 rpm, 15 min) (MPW-352, MPW MED. INSTRUMENTS, Warsaw, Poland), separating the solids from the filtrate. The filtrate was concentrated at 70 °C to about half the original volume. The thus-concentrated solution was crystallized by cooling (5 °C), and the crystals of purified FeSO_4_ were separated from the filtrate. The performed process is illustrated in the schematic diagram in Figure 1.

### 2.3. Preparation of Iron Pigments

The effect of the concentration of starting solutions on the synthesis of iron pigments was investigated. For this purpose, salt solutions with concentrations of 10, 14, 21, and 28 wt% of purified FeSO_4_ were prepared. Accordingly, the names of the synthesized pigment samples were introduced: CZ 10%, CZ 14%, CZ 21%, and CZ 28%.

First, the assumed amounts of purified FeSO_4_·7H_2_O (7.5 g; 10 g; 15 g; 20 g) were stirred at room temperature until they formed a transparent solution in aqua (~15 mL). For the oxidation, stoichiometric amounts of hydrogen peroxide (30 wt%) were used (1).

(1) $$2{\mathrm{FeSO}}_{4}+H_{2}O_{2}+H_{2}{\mathrm{SO}}_{4}\to {\mathrm{Fe}}_{2}{\left({\mathrm{SO}}_{4}\right)}_{3}+2H_{2}O$$

Then, to precipitate the iron hydroxide, 25 wt% ammonia water was added in stoichiometric amounts (2).

(2) $${\mathrm{Fe}}_{2}{\left({\mathrm{SO}}_{4}\right)}_{3}+6{\mathrm{NH}}_{3}\cdot H_{2}O\to 2\mathrm{Fe}{\left(\mathrm{OH}\right)}_{3}↓+3{\left({\mathrm{NH}}_{4}\right)}_{2}{\mathrm{SO}}_{4}$$

Suspension was then transferred into a Teflon container, filled with water to a volume of 70 mL (Teflon container volume 100 mL), and placed in the microwave reactor (Ertec Magnum II) where the reaction was carried out for one hour at a pressure range of 17–20 bar and a temperature of 170 °C. At the end of the period, the resulting suspensions were washed with water and dried at 105 °C for 4 h.

### 2.4. Analytical Methods

The chemical composition of waste and purified FeSO_4_ was determined by quantitative analysis with the ICP-OES method (Perkin Elmer Avio 500, Waltham, MA, USA). The content of Fe(II) and Fe(III) was quantified by manganometric titration [31]. The amount of waste for testing was secured according to PN-EN 1482-1:2008 and PN-EN 1482-3:2016-09. XRD patterns were collected with an X-ray diffractometer (Empyrean, Malvern Panalytical, Malvern, UK) equipped with a Cu–Kα radiation source (Nickel filter; λ = 0.15418 nm, 40 kV, 35 mA). Scans were taken at room temperature in scattering 2θ range of 10–100° with a step interval of about 0.026°. Phase composition was determined using Panalytical X’Pert HighScore Plus v3.0 software with the ICDD PDF4+ database. Fourier transform infrared spectroscopy (FTIR) spectra of the sample were measured in a range of 4000–400 cm^−1^ on a Nicolet 380 spectrometer (Thermo Fisher Scientific, Waltham, MA, USA) and the sample was mixed with KBr at a ratio of 1:100 and then compressed into tablets. The surface composition of pigments was analyzed with X-ray photoelectron spectroscopy (XPS). The photoelectron measurements were conducted with Mg Ka (hν = 1253.6 eV) radiation in a Prevac system equipped with Scienta SES 2002 electron energy analyzer operating at constant transmission energy (Ep = 50 eV). The pressure in the analysis chamber was kept under 1∙10^−9^ mbar. The specific surface area of pigments was determined by the Brunauer–Emmett–Teller (BET) method using the Quadrasorb Evo Quantachrome Instruments nitrogen adsorption apparatus. A sample was degassed at 100 °C under a high vacuum for 16 h. Dynamic light scattering measurements (DLS) were carried out on the Horiba LA 950 Laser Diffraction Particle Size Analyzer. A sample was dispersed in 50 mL of 0.1% sodium pyrophosphate solution. Then, the solution was sonicated in an ultrasonic cleaner for 2 min. The prepared dispersion was loaded into the analyzer. In the measurements, a reflectance coefficient of 2.90 was used, and sonication was turned on. The surface morphology of samples was observed with an emission scanning electron microscope VEGA 3 (TESCAN, Brno, Czech Republic). A sample was dispersed in isopropyl alcohol, and 2 µL were placed on silicon wafers and evaporated. Oil absorption was determined according to the PN-EN ISO 787-5:1999 standard. 

## 3. Results and Discussion

Table 1 presents the chemical composition of the salt, determined by the ICP-OES method. The content of iron ions was determined by manganometric titration. Besides iron(II) and(III) ions, the waste FeSO_4_ contained admixtures of other metals in the form of ions such as titanium, magnesium, calcium, potassium, sodium, manganese, and others.

The content of a significant part of Fe^3+^ ions (8.7 + −0.2 wt%) proves that the Fe^2+^ ions contained in the waste FeSO_4_, as a result of long-term storage, were oxidized by atmospheric air. In addition, high levels of magnesium and titanium indicate residues of undecomposed titanium ore. The purification process allowed for a decrease in the content of elements such as Mg, Ca, Na, Zn, and Ti. Recrystallization also increased the content of Fe^2+^ ions in a sample, which is also a positive effect of this process. During the removal of impurities, iron ions are also removed to a small extent. The iron losses are insignificant considering how low the contaminants level achieved after purification is.

**Figure 2:**
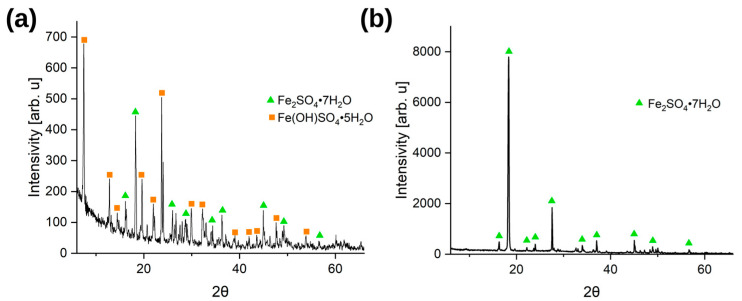
XRPD pattern of (**a**) waste FeSO_4_, (**b**) purified FeSO_4_.

Figure 2 shows the diffractograms of (a) waste FeSO_4_ and (b) purified FeSO_4_. Two phases of iron(II) sulfate heptahydrate (ICDD No.: 04-010-4265) and iron(III) hydroxide sulfate pentahydrate (ICDD No.: 00-016-0935) were identified in the waste material, which is marked in the diffraction pattern. As a result of the recrystallization process, a salt was obtained in which one crystalline phase was identified, derived from iron(II) sulfate heptahydrate (ICDD No.: 04-010-4265).

So far, attempts have been made to develop a method for treating waste long-deposited iron(II) sulfate. However, these methods either use fresh salt with low amounts of impurities and Fe^3+^ [32] or rely on complex chemical reactions, taking into account redox reactions or impurity precipitation [33,34]. These methods are much more complicated compared to the method described in the article.

### 3.1. Iron Pigment Characterization

#### 3.1.1. XRD

**Figure 3:**
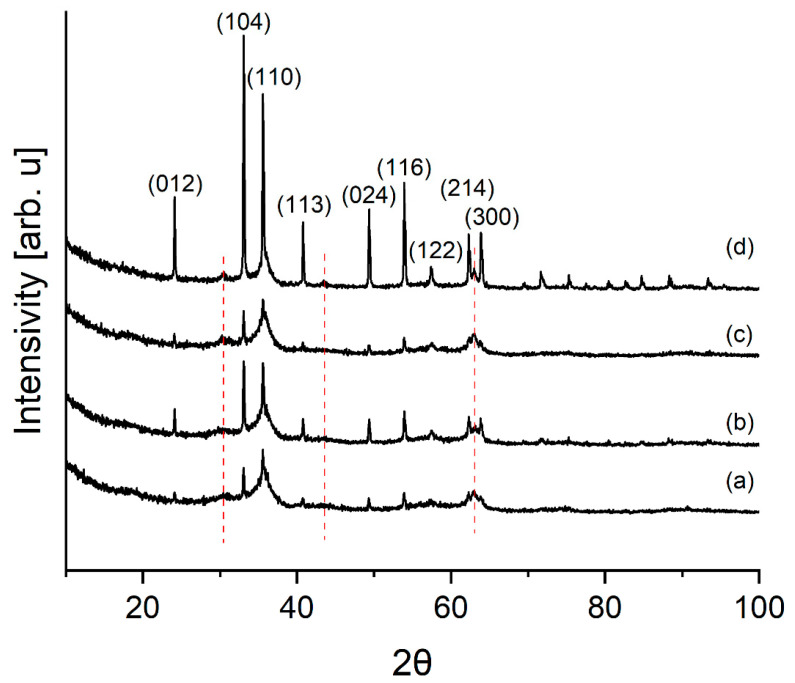
XRPD pattern of laboratory pigment samples: (**a**) CZ 10%, (**b**) CZ 21%, (**c**) CZ 28%, (**d**) CZ 14%. Red lines mark the remnants of geothite.

Figure 3 shows X-ray powder diffraction (XRPD) diffractograms of the laboratory pigments.

As can be seen, the samples consist of hematite (ICDD No.: 00-024-0072). Samples contain, in addition to partially crystallized compounds, a significant amount of the amorphous phase. The broad bases of the reflection (110) suggest that hematite was formed by dehydration of goethite [35]. Red lines indicate places where goethite remnants can be seen in the diffraction pattern (ICDD No.: 01-073-8431).

**Figure 4:**
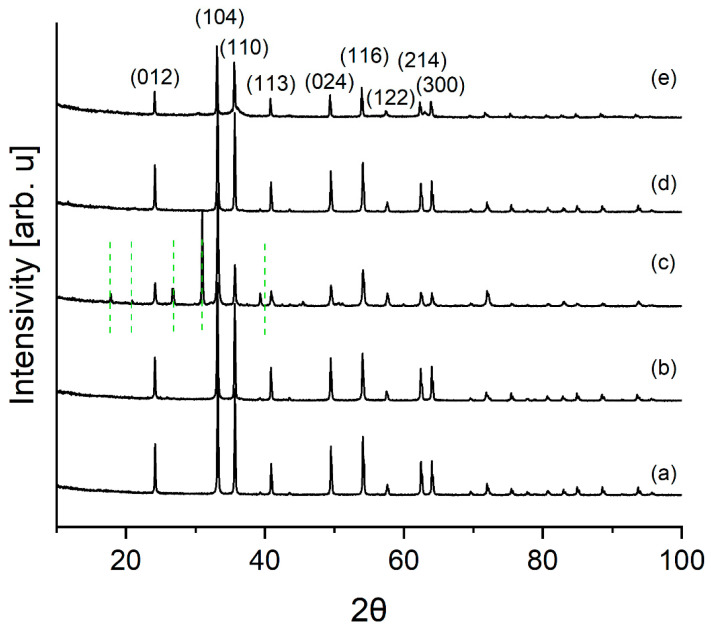
XRPD pattern of iron pigment samples: (**a**) Commercial pigment A, (**b**) Commercial pigment B, (**c**) Commercial pigment C, (**d**) Commercial pigment D, (**e**) CZ 14%. Green lines mark the content of SiO_2_ phase.

Figure 4 shows the diffraction patterns of commercial pigments and the pigment *CZ 14%.*

The diffraction patterns of commercial pigments show reflections characteristic of the hematite phase (ICDD No.: 00-024-0072). In addition to hematite, the reflections characteristic of silica (green lines) (ICDD No.: 01-080-2148) are visible in the *Commercial pigment C* diffraction pattern.

#### 3.1.2. FT-IR

**Figure 5:**
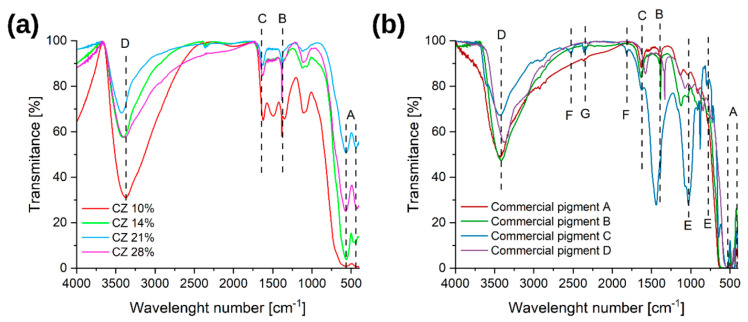
FTIR spectra for (**a**) laboratory pigments; (**b**) commercial pigments. A–G are the functional group designations.

On the obtained FT-IR spectra (Figure 5) can be observed a band of vibrations typical of iron oxides from the Fe–O bond A [11,36,37,38] at positions of about 480 cm^−1^ and 560 cm^−1^. The next characteristic bands appear at positions 800 cm^−1^, 900–990 cm^−1^, and 1450 cm^−1^ and they correspond to the vibrations typical of the O–H bond B [11,36]. The band C is characteristic of the bending vibrations of the O–H groups [11,37]. The last, wide band with a maximum at the position of about 3400 cm^−1^ is representative of the water absorbed on the sample surface, and more specifically for the stretching vibrations of the O–H groups D [11,37,38,39]. According to the literature, the higher the crystallization of the materials, the greater the shift of the characteristic bands that is observed [36].

In commercial pigments, apart from the characteristic bands described earlier, in one sample there are also other compounds. Thus, for the sample of *Commercial pigment C*, at the values of about 750 cm^−1^ and 1100 cm^−1^, there is a band characteristic of the Si–O bond E [40]. Then, at the value of about 1530 cm^−1^, vibrations originating from the Ca–O bond F [41] occur, and at about 2500 cm^−1^, a low-intensity band G [42,43], originating from CO_2_, appears. This indicates the presence, in addition to silica, of calcite (CaCO_3_), which is a fairly popular surface treatment agent.

#### 3.1.3. XPS

**Figure 6:**
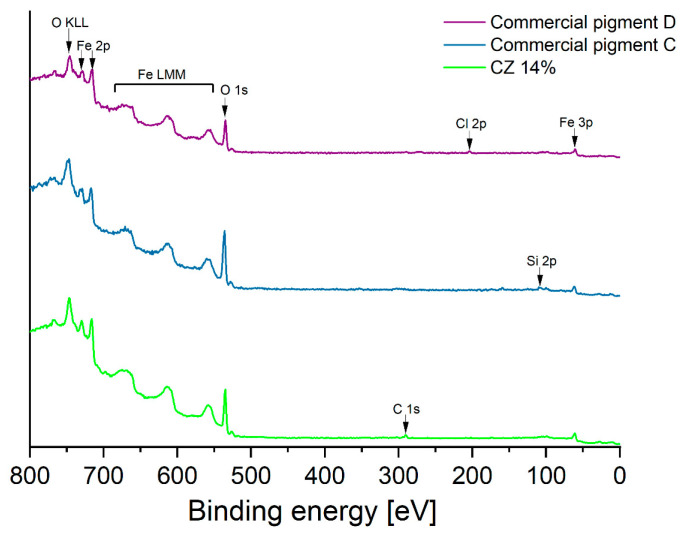
The survey XPS spectra of CZ 14% and Commercial pigments C and D.

The surface of *CZ 14%* pigment as well as those of *Commercial pigments C* and *D* were analyzed by X-ray photoelectron spectroscopy (Figure 6). These tests showed that the surfaces of all three materials contain predominantly oxygen atoms and iron atoms from iron oxides, the main component of pigments. The only contamination on the surface of the *CZ 14%* pigment is “adventitious carbon”, usually occurring on the surface of materials prepared by wet synthesis. Analysis of the surface of *Commercial pigment C* confirms the conclusions of diffractometric methods and FTIR analysis, i.e., traces of silicon and calcium atoms, presumably from oxides of these metals, is observed on the surface. Traces of chlorine were identified on the surface of *Commercial pigment D*. 

**Figure 7:**
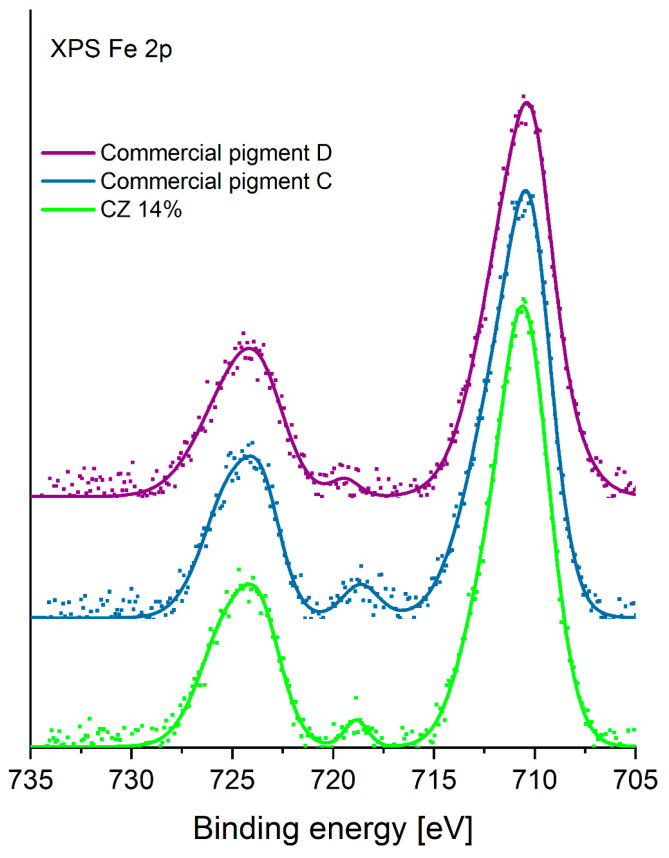
The XPS Fe 2p spectra of CZ 14% and Commercial pigments C and D.

A detailed analysis of the chemical state of iron in the three mentioned pigments was also performed. The XPS spectrum of Fe 2p is shown in Figure 7. This analysis shows that the chemical state of iron in all the analyzed pigments is identical, indicating the presence of the same iron compounds. XPS analysis of complex iron–oxygen compounds often does not allow a conclusive distinction of the chemical states of iron present in the material under study. This is particularly difficult in the case of the presence of a mixture of iron oxides. In the present study, by comparing the shape of the Fe 2p line envelope, it was determined that the material most likely contains Fe^3+^ iron ions [44].

#### 3.1.4. Determination of the Specific Surface by the BET Method

**Figure 8:**
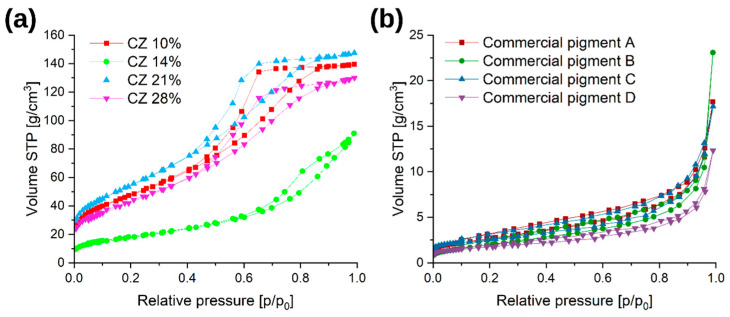
Results obtained from BET analysis: (**a**) nitrogen adsorption isotherms of laboratory pigments; (**b**) nitrogen adsorption isotherm of commercial pigments.

Figure 8 shows nitrogen adsorption and desorption isotherms for laboratory and commercial pigments.

Laboratory pigments have an isothermal shape that corresponds to type V according to the IUPAC classification [45,46]. This is a typical characteristic of mesoporous materials. The hysteresis shape corresponds to the E type, i.e., spherically elongated or bottle-shaped pores with open ends. The shape of the adsorption isotherms for commercial pigments also corresponds to isotherms for mesoporous materials, but with a much lower adsorption capacity.

**Figure 9:**
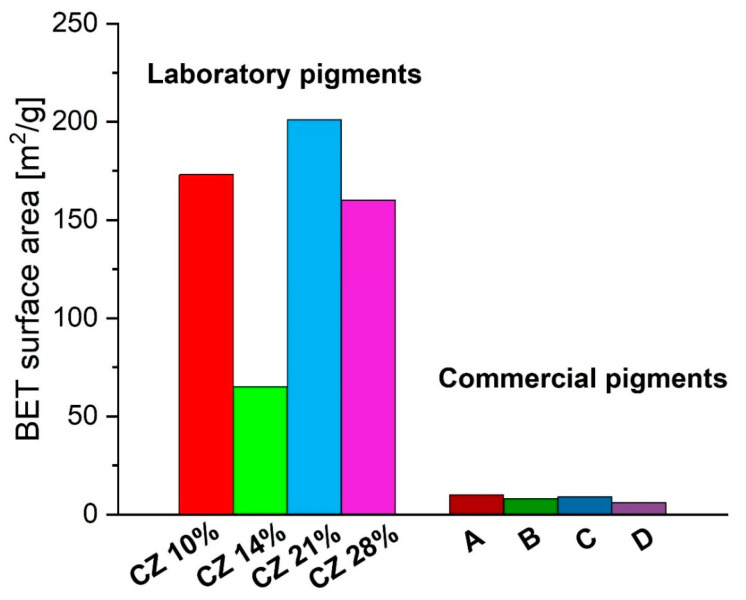
BET surface area of laboratory and commercial pigments.

Synthesized pigments have up to 20 times greater specific surface area than commercial pigments (Figure 9). The differences in results can be explained by the method of sample synthesis—commercial pigments obtain their proper structure at the stage of high-temperature calcination [1,11,47]. The temperature of the goethite–hematite transformation is 450 °C, while during calcination the temperature is much higher and reaches over 600 °C. That difference promotes the sintering of the pigment and the disappearance of the developed specific surface. In the literature [48,49], hematite obtained at a temperature of 450 °C has a specific surface area in the range of 25–40 m^2^/g, which is 3–4 times larger than the characterized commercial iron pigments. In the case of synthesized pigments, it is visible that the higher the amorphous phase content in the samples, the higher the specific surface area of the material. Sample *CZ 14%*, containing well-crystallized hematite, shows a value of the specific surface area close to the value in the literature [48,49].

As per the definition proposed by the European Commission [50], all obtained laboratory materials that have a surface area of more than 60 m^2^/cm^3^ can be considered “nanomaterials”.

#### 3.1.5. DLS

**Figure 10:**
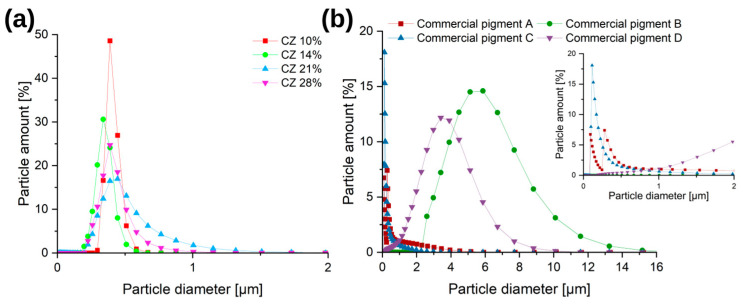
Pigment size distribution analysis by dynamic light scattering of (**a**) laboratory and (**b**) commercial materials.

Figure 10 shows the particle size distribution for laboratory and commercial pigments.

Most of the commercial pigments presented here have a wide particle size distribution. In *Commercial pigment A* and *Commercial pigment C* samples, the majority of the particles are in the range of 0–500 nm with a small proportion (<10%) of larger particles. The rest of the commercial pigments show primarily particles in the 2–9 µm range, with a small proportion (<10%) of particles smaller than 2 µm or larger than 9 µm. Due to the sonication of the suspension during the measurements, particle sedimentation can be excluded [11,51].

In the case of laboratory pigments, the obtained materials were mostly characterized by a particle distribution in the range of 0.1–1 µm. The *CZ 14%* and *CZ 10%* samples had the highest proportion of particles in the 100–500 nm range; the proportion of particles of this size in these samples was circa 90%.

#### 3.1.6. Pigment Observation on SEM

**Figure 11:**
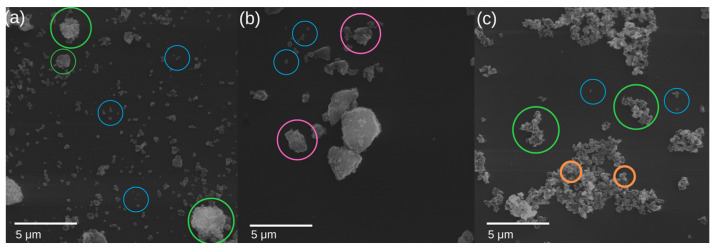
SEM images of iron pigments (SEM HV: 30 kV; SEM MAG: 30 kx): (**a**) CZ 14%, (**b**) CZ 28%, (**c**) Commercial pigment A.

Figure 11 shows SEM photos.

Laboratory pigments do not show such strong agglomeration. In the case of the *CZ 14*% sample, the photos show individual plates about 300 nm in size, which is consistent with the results of the DLS analysis. Several aggregates of particles are also observed. In the case of the *CZ 28*% sample, the pigment structure is not as distinguishable as in the case of the *CZ 14*% sample. The photos show agglomerates of particles. Blue particles are single particles up to 500 nm in size. Agglomerates of particles > 500 nm with single particles highlighted are green. Orange granules are individual particles forming agglomerates with a size of 100–600 nm. Pink particles are aggregates of particles < 2µm with indistinguishable single particles. Particles are irregular with spherical shape [11].

#### 3.1.7. Oil Absorption

**Figure 12:**
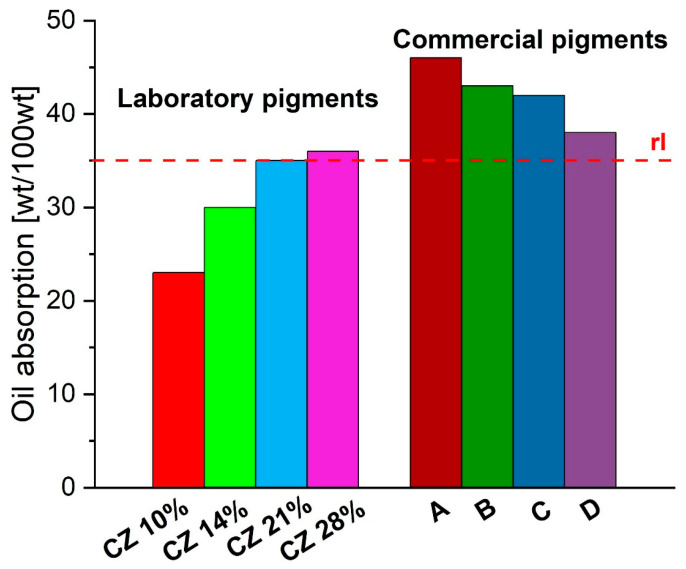
Oil absorption for laboratory and commercial pigments (rl—reference level).

According to the technical sheets that were obtained regarding the product, each of the commercial pigments should have an oil absorption of a maximum of 35 g/100 g (rl) based on the product technical sheets. All of the values for the commercial pigments exceed 10 to 30 percent of the reference values (Figure 12). For laboratory materials, pigments synthesized at low concentrations are characterized by lower values of the oil absorption than pigments synthesized at a concentration > 20 wt%.

## 4. Conclusions

This study developed a purification method for waste FeSO_4_, and then a method for synthesizing red iron pigments using purified FeSO_4_. Results of the study indicate that recrystallization is an effective method of purifying waste long-term storage iron(II) sulfate, allowing the iron(II) content to increase while reducing the impurities—especially coloring compounds, which affect the pigment color (Mn compounds). In addition, the developed purification method does not require complicated equipment or many chemical reagents.

XRD measurements of the pigment samples from different concentrations confirm that the prepared nanoparticles consist mostly of hematite phase, also supported by FTIR results. Results showed that the pigment synthesized from a solution of 14% iron salt had the most crystallized hematite when compared to other pigments. This may be related to the suspension:total reactor volume ratio and the fact that the growth of hematite crystals is controlled only by kinetic factors. In the coprecipitation process, two stages are involved: a short burst of nucleation occurs when the concentration of the species reaches critical supersaturation, and then there is a slow growth of crystals [52,53]. The optimal concentration of iron sulfate based on research can be 10–20% (49–92 mM) with an optimum of 14 wt% (66 mM).

In addition, synthesis using the microwave reactor is reproducible. Laboratory pigments do not show strong agglomeration. The *CZ 14%* sample shows individual plates about 300 nm in size, while the *CZ 28%* sample shows agglomerates of particles. The SEM study found that laboratory pigment particles aggregate less than commercial pigments. In addition, synthesized pigments have some irregular spherical shapes. High surface area (>60 m^2^/g) of the nanoparticles synthesized with mesoporous structure was performed by BET. In addition, the XRD and XPS analyses revealed the purity of the hematite nanoparticles. The oil absorption of most pigments synthesized in the laboratory was in the range of 23–39 g/100 g of pigment and was in the range declared for commercial pigments (max. 35 g/100 g).

Based on the obtained findings, it can be concluded that waste long-term storage iron(II) sulfate is a potential raw material for the production of iron pigments. On this basis, pigments are obtained with physicochemical properties comparable to commercially available pigments, and their suggested use is in the dyeing industry.

## Figures and Tables

**Table 1:** Content of elements in waste and purified FeSO_4_.

	Fe^2+^	∑Fe	Mg	Ti	Ca	K	Mn	Zn	Ni	Cr
	(wt%)			(ppm)						
Waste FeSO_4_	9.2 ^(a)^	17.9 ^(a)^	5.97	11,544	1322	718	513	404	392	<dl ^(b)^
Purified FeSO_4_	14.2 ^(a)^	16.2 ^(a)^	0.68	20	347	64	189	205	38	<dl ^(b)^

^(a)^ The content of iron ions was determined by manganometric titration; ^(b)^ detection limit.

## Data Availability

The data presented in this study are available on request from the corresponding author.
